# Cell death and senescence

**DOI:** 10.1186/s12967-023-04297-y

**Published:** 2023-06-29

**Authors:** Lorenzo Galluzzi, Melissa Myint

**Affiliations:** 1 Department of Radiation Oncology, Weill Cornell Medical College, New York, NY USA; 2 Sandra and Edward Meyer Cancer Center, New York, NY USA; 3 Caryl and Israel Englander Institute for Precision Medicine, New York, NY USA; 4 Sonata Therapeutics, Watertown, MA USA

## Main text

We are delighted to announce the launch of a new section of the *Journal of Translational Medicine* on “Cell Death and Senescence”.

Eukaryotic cells exposed to extreme perturbations of homeostasis, which are relatively rare in nature, succumb to the unregulated and virtually immediate physical breakdown of their components, a process that has been dubbed accidental cell death (ACD) [1]. Most often, however, eukaryotic cells are exposed to relatively mild perturbations of their microenvironment, which results in the activation of stress-responsive pathways that are in place to repair macromolecular damage and recover physiological cellular functions [2, 3]. These mechanisms encompass, but are not limited to: the DNA damage response [4], the unfolded protein response [5], and autophagy [6]. When cellular damage can be efficiently repaired and/or microenvironmental perturbations are limited in intensity and duration, cells can recover physiological functions in the context of re-established homeostasis [2, 3]. On the contrary, when damage is beyond repair and/or stressful stimuli are excessively intense or prolonged, the same pathways that initially attempt to restore physiological homeostasis instead engage signaling modules that actively promote cellular demise, a process that has been dubbed regulated cell death (RCD) [1].

As it stands, a number for different RCD modalities has been defined based on key biochemical events [7]. These RCD routines include (but are not limited to): (1) extrinsic and intrinsic apoptosis: two RCD modes involving the activation of proteases of the caspase family that are initiated by perturbation of extracellular and intracellular homeostasis, respectively, with the latter being demarcated by mitochondrial outer membrane permeabilization (MOMP) [8, 9]; (2) mitochondrial permeability transition (MPT)-driven regulated necrosis, a form of RCD initiating with the rapid permeabilization of the inner mitochondrial membrane via a mechanism that involves peptidylprolyl isomerase F (PPIF, best known as CYPD) [10]; (3) necroptosis, a type of regulated necrosis that relies on a signaling core platform involving receptor interacting serine/threonine kinase 3 (RIPK3) and mixed lineage kinase domain like pseudokinase (MLKL) [11]; (4) ferroptosis, an iron-dependent RCD modality that is under tonic inhibition by glutathione peroxidase 4 (GPX4) [12]; and (5) pyroptosis, a variant of necrotic RCD that is demarcated by plasma membrane permeabilization as driven by gasdermin D (GSDMD) or gasdermin E (GSDME) [13, 14].

Importantly, most if not all RCD modalities exhibit a considerable degree of interconnectivity [15], which implies that inhibiting specific components of the system generally delays RCD (and changes its morphological and immunological correlates) but does not prevent it altogether [1]. Moreover, it has now become clear that multiple biochemical mechanisms that were initially considered as the actual drivers of RCD, such as the post-MOMP activation of caspase 3 (CASP3), only control the kinetics of RCD and the interaction of dying cells with the host, but do not determine whether or not RCD will ultimately occur [1, 16]. Intriguingly, such an interaction, which largely (but not exclusively) involves the host immune system, does not emerge only once cells have irremediably committed to death but actually much earlier, during early adaptation to stress (irrespective of whether this will ultimately be successful or not) [17, 18].

Of note, RCD is not the sole mechanism through which multicellular organisms control individual cells that are damaged beyond repair and hence cannot fulfill their functions and perhaps even be dangerous as potentially tumorigenic [3]. Indeed, when eukaryotic cells accumulate somehow intermediate degrees of macromolecular damage, which cannot be efficiently repaired but also do not actively engage RCD, a permanent proliferative arrest associated with a considerable shift in the cellular secretome occurs [19, 20]. This process, which has been dubbed cellular senescence, resembles RCD in that it can also be elicited by perturbations of intracellular or extracellular homeostasis [21]. However, while all senescence inducers cause an irreversible proliferative arrest, the so-called “senescence-associated secretory phenotype” (SASP) exhibits considerable degrees of context dependency [21].

Importantly, dysfunctions in the molecular and cellular mechanisms through which individual eukaryotic cells respond to stress (either successfully or not) and interact with their host in the process, including (but not limited to) the DNA damage response, the unfolded protein response, autophagy, RCD and cellular senescence have been attributed pathological significance in a plethora of human disorders [9, 22]. The new *Journal of Translational Medicine* section on “Cell Death and Senescence” now opens to consider original contributions, review articles and editorials discussing mechanistic and pathophysiological aspects of all these processes.

The *Journal of Translational Medicine* is committed to providing authors with rapid editorial decisions, not only as novel incoming contributions are evaluated for suitability, novelty, and scientific value by Section and Associate Editors, but also when expert scientists return their criticism as part of the peer-reviewing process. The new section on “Cell Death and Senescence” will embrace this mission to guarantee high quality and competitive publications, and its Editorial Board is very much looking forward to receiving your contributions.

## Data Availability

Not applicable.
